# Does Inguinal TAPP Repair Increase the Rate of Midline Supraumbilical Trocar Site Hernia?—A Single-Center Retrospective Study †

**DOI:** 10.3390/jcm15083083

**Published:** 2026-04-17

**Authors:** Goran Augustin, Karmen Jeričević, Branko Bogdanić

**Affiliations:** 1 Deparment of Surgery, University Hospital Centre Zagreb, 10000 Zagreb, Croatia; karmen.jericevic@kbc-zagreb.hr (K.J.); bbogdani@kbc-zagreb.hr (B.B.); 2 School of Medicine, University of Zagreb, 10000 Zagreb, Croatia

**Keywords:** inguinal hernia, recurrence, TAPP, trocar site hernia, umbilical hernia

## Abstract

**Background/Objectives**: This study aimed to determine the inguinal hernia recurrence rate after transabdominal preperitoneal (TAPP) repair, particularly considering the effect of simultaneous umbilical hernia repair. The secondary aim was to assess whether closing the 10 mm midline supraumbilical port-site fascia affects the incidence of trocar-site hernia (TSH) following inguinal TAPP. **Methods**: We reviewed medical records of consecutive patients undergoing inguinal TAPP at the Department of Surgery, University Hospital Centre Zagreb, between 1 January 2014 and 30 June 2022, and supplemented the data with telephone follow-up. Demographic, clinical, and operative variables were compared between patients who did and did not report inguinal hernia recurrence. Patients were also grouped by operating surgeon to compare TSH rates. Surgeon A routinely closed the 10 mm supraumbilical fascial defect with a single suture, while Surgeon B mostly did not, deciding on a case-by-case basis. **Results:** The analysis included 281 patients with a median follow-up of 60 months. The overall recurrence rate was 10.6%. Baseline demographic and clinical characteristics did not differ significantly between patients who reported recurrence and those who did not. A prior hernia repair was more common in the recurrence group (34.1% vs. 17.2%; *p* = 0.007). Concomitant umbilical hernia repair was performed in 12.5% of cases. Patient-reported recurrence was higher after combined TAPP and umbilical hernioplasty than after TAPP alone (14.3% vs. 12.2%), but this difference was not statistically significant (*p* = 0.784). Surgeon A had a lower observed TSH rate than Surgeon B (1.0% vs. 3.6%), although this difference did not reach statistical significance (*p* = 0.242). **Conclusions**: Concurrent TAPP and umbilical hernioplasty is not associated with a higher recurrence rate, but further research on a larger cohort is necessary. Routine closure of the 10 mm midline supraumbilical fascial defect could reduce the TSH rate, although the difference was not statistically significant. The side of the hernia does not influence recurrence after TAPP.

## 1. Introduction

Umbilical and inguinal hernias are common conditions requiring surgery. Their frequency is rising due to a growing global population and increased longevity. In 2019, there were 32.53 million existing cases and 13.02 million new cases of inguinal, femoral, and abdominal hernias worldwide. This represents a significant increase of 36% and 64%, respectively, compared to 1990 [1]. The occurrence is higher in older males, peaking in the 65–69 age group. Projections through 2030 suggest that hernia prevalence will keep increasing, mainly among men, even though age-standardized mortality and DALY rates are expected to decrease [2].

Although open mesh repair, also known as the Lichtenstein technique, remains the gold standard, the use of minimally invasive approaches is increasing rapidly. The most common minimally invasive procedures, transabdominal preperitoneal (TAPP) and totally extraperitoneal (TEP) repair, accounted for a third of primary groin hernia repairs between 2004 and 2020 [3]. The HerniaSurge Group international guidelines recommend a tailored approach for the management of inguinal hernia, emphasizing that no single surgical technique is suitable for all patients. Laparo-endoscopic techniques, including TAPP and TEP, are supported as evidence-based options for primary unilateral inguinal hernia repair, particularly when adequate surgical expertise and resources are available [4,5]. This recommendation is based on lower postoperative and chronic pain compared to the Lichtenstein repair [5]. Results after TAPP repair show significantly less chronic inguinal pain than after the Lichtenstein repair, without significant differences in other outcomes such as postoperative complications and hernia recurrence [6]. Some authors report recurrence rates as low as 1.1% on a 1-year follow-up [7], though higher rates have been observed with longer follow-up [8].

Trocar site hernia (TSH) is a type of incisional hernia that develops at the sites where trocars are inserted. It was first identified in 1991 after a laparoscopic cholecystectomy [9]. The overall occurrence following laparoscopic surgery is reported to range from 0% to 5.2%, particularly with 10 mm or larger trocars. A higher rate was observed when the fascia at the trocar site was not sutured [10]. For TSH in the umbilical region after the TAPP procedure, the overall rate is 3.9% [11]. Guidelines recommend suturing the fascial defect at trocar sites measuring 10 mm or more, although limited data support the necessity of fascial closure [12].

A lack of long-term follow-up after the TAPP procedure still exists, especially among diverse patient groups that include both men and women, patients with unilateral or bilateral inguinal hernias, and those with recurrent (previously repaired) hernias. Additionally, TSH in this group often requires a second surgery and has not been thoroughly studied. We found only one other study analyzing TSH in the umbilical region after the TAPP procedure, but it had a shorter follow-up of 20 months and included patients who underwent simultaneous umbilical hernia repair [11].

The main goal of this study was to identify surgical risk factors for inguinal hernia recurrence after TAPP, especially when performed alongside umbilical hernia repair. Additionally, TSH rates and their related risk factors were examined in patients without a history of umbilical hernia.

## 2. Materials and Methods

### 2.1. Patients

The data were collected from electronic and paper medical records, as well as telephone interviews with consecutive patients admitted for TAPP hernia repair at the Department of Surgery at the University Hospital Centre (UHC) Zagreb, between 1 January 2014 and 30 June 2022. We used a structured questionnaire specifically designed for this study (Supplementary Table S1). Telephone interviews were conducted from September 2023 to July 2025.

### 2.2. Ethical Considerations

The study received approval from the institutional ethics committee of UHC Zagreb (Class 8.1-25/166-2, Number 02/013 AG, Date 25 July 2025). The consent form for participation was distributed to all participants and signed at the time of initial hospitalization. The consent form included consent for evaluation during follow-up.

### 2.3. Study Design

Electronic and paper medical records were reviewed for demographic data (age, gender), clinical information (BMI, ASA score, comorbidities such as smoking and diabetes), previous hernia surgeries (umbilical and inguinal), and surgical details (type of TAPP procedure, whether bilateral or unilateral, as well as concurrent umbilical hernia repair). Postoperative complications assessed included seroma or hematoma, shoulder pain, groin pain, and abdominal pain. The remaining data were collected during telephone interviews, which covered hernia recurrence, time to return to work or usual daily activities for unemployed or retired patients, physical activity levels, and family history of hernia. During these interviews, patients were asked about postoperative complications and physical activity levels using the occupational activity classification proposed by Steeves et al. [13]. A positive family history was defined as having a parent or sibling with an inguinal hernia (Supplementary Table S2).

During the laparoscopic procedure, both inguinal regions were examined for hernia, even if only one side was operated on. Exclusion criteria included pediatric patients (age 16 and under), emergency admissions (such as incarcerated hernias), femoral hernias, and patients unable to determine if they experienced a recurrence. Telephone interviews were attempted at least twice over a 2-week period for all patients.

### 2.4. Surgical Technique

Both surgeons had similar clinical experience, having performed more than 100 cases each. For umbilical hernias, both surgeons first dissected the hernia, inserted a 10 mm trocar through the defect, and placed sutures around the defect to prevent loss of pneumoperitoneum. The other two 5 mm trocars were placed in standard locations. A 15 cm × 15 cm polypropylene mesh was cut to the appropriate shape and secured with titanium tackers. The peritoneum was also closed with tackers.

Surgeon A consistently used fascial closure of the 10 mm supraumbilical port with a single absorbable (polyglactin) suture 0 and a 5/8 round needle; Surgeon B decided on a case-by-case basis, but mostly did not perform fascial closure.

### 2.5. Study Outcomes

In the first part of the study, patients were divided into two groups: those who reported hernia recurrence and those who did not. In the second part, patients were categorized by surgeon.

The primary goal was to compare the demographic, clinical, and surgical characteristics of the two groups (recurrence and non-recurrence) and to evaluate surgical risk factors for recurrence. The secondary goal was to compare the incidence of midline supraumbilical TSH after the TAPP procedure between the two surgeons, who used different port-closure techniques. TSH was defined as a new incisional hernia at the 10 mm supraumbilical trocar site in patients without a pre-existing umbilical hernia.

### 2.6. Statistical Analysis

All data were entered into the EXCEL database (Microsoft, Washington, DC, USA). The statistical analysis was performed using SPSS version 29.0.2.0 (SPSS, Inc., Chicago, IL, USA). The normality of the distribution for quantitative variables was assessed with the Shapiro–Wilk test. Continuous data are presented as medians with interquartile ranges (IQR) and compared between groups using the Mann–Whitney U test. Ordinal data are shown as absolute numbers and percentages and compared using the Mann–Whitney U test. Categorical data are reported as counts with percentages and compared between groups using the χ^2^ (chi-square) test or Fisher’s exact test, as appropriate. Ninety-five percent confidence intervals (95% CI) for proportions and differences in proportions were calculated using the Wilson score and Newcombe (Wilson-based) methods, respectively. All tests were two-sided, and *p*-values < 0.05 were considered statistically significant.

To identify factors independently associated with recurrence, an additional multivariable logistic regression analysis was performed at the patient level, using patient-reported inguinal hernia recurrence during follow-up as the dependent variable. Due to the limited number of recurrence events, a parsimonious model was built that included clinically relevant covariates available in the dataset: age, sex, body mass index (BMI), history of previous hernia repair, bilaterality of the index TAPP procedure, and operating surgeon. Results are presented as adjusted odds ratios (aORs) with 95% confidence intervals (CIs). A sensitivity analysis was also performed, including concomitant umbilical hernia repair. A two-sided *p*-value < 0.05 was considered statistically significant.

## 3. Results

During the study period, 394 adult patients admitted to the Department of Surgery at UHC Zagreb for TAPP inguinal hernia repair were eligible. Two surgeons, Surgeon A and Surgeon B, performed surgeries on 334 (84.8%) patients, while other surgeons operated on 60 (15.2%) patients. The statistical analysis focused on the two surgeons who performed the most TAPP procedures. Of those 334 patients, 39 (11.7%) did not respond to attempted phone interviews, 13 patients (3.9%) passed away, and one patient (0.3%) could not report a recurrence.

Overall, 281 patients were included (Table 1). Patient-reported recurrence occurred in 12.5% of patients. The median follow-up was 59.0 months for the recurrence group and 61.0 months for the no-recurrence group (*p* = 0.895). There was no significant difference in age, sex, BMI, comorbidities, distribution of ASA scores, family history of hernia, or physical activity level (Table 2). Smoking was present in 20.0% of patients with recurrence and in 21.5% of those without recurrence; a difference of −1.5 percentage points (pp; 95% CI −17.1 to 19.0; *p* = 0.835). In the recurrence group, 8.6% of patients had diabetes compared to 5.3% in the no-recurrence group; a difference of 3.3 pp (95% CI −5.9 to 19.3; *p* = 0.431). A positive family history was present in 34.3% of patients with recurrence vs. 29.3% without recurrence; a difference of 5.0 pp (95% CI −11.7 to 21.7; *p* = 0.560).

A bilateral TAPP was performed in 136 patients (48.4%), while a unilateral TAPP was done in 145 patients: 87 (31.0%) on the right side and 58 (20.6%) on the left side (Table 3). The side of the inguinal hernia does not influence recurrence after TAPP repair (*p* = 0.827).

The median time to resume work or usual daily activities was three weeks, with no statistically significant difference between the groups (Table 4). The most common complications were seromas and hematomas, occurring in 52 (18.5%) patients. Less common were shoulder, groin, and abdominal pain. There was no significant difference in complication rates between the groups.

Among the 281 patients, 417 inguinal hernias were identified (Table 5), with 44 (10.6%) patients reporting hernia recurrences. There was no significant difference in the recurrence rate between the right and left sides: 11.7% vs. 9.3%; a difference of 2.4 pp (95% CI −6.1 to 10.6); *p* = 0.430.

TAPP was performed on 79 previously repaired (PR) hernias out of 417 cases (18.9%; Table 6). The recurrence group showed a higher proportion of PR hernias compared to the no recurrence group: 34.1% vs. 17.2%; a difference of 16.9 pp (95% CI 2.4 to 31.5); *p* = 0.007. The same trend was observed in right- and left-sided PR hernia subgroups, with a significant difference noted in the right-sided group. There were 42.3% right-sided PR hernias in the recurrence group compared to 18.8% in the no recurrence group; a difference of 23.5 pp (95% CI 0.8 to 47.1); *p* = 0.006. For left-sided hernias, PR hernias accounted for 22.2% in the recurrence group vs. 15.3% in the no recurrence group; a difference of 6.9 pp (95% CI −12.4 to 34.4); *p* = 0.497.

Umbilical hernia was present in 36 (12.8%) patients before the TAPP procedure, and 35 (12.5%) underwent umbilical hernia repair during the TAPP procedure; in one patient, the umbilical hernia was not repaired (Table 7). For the analysis of the TSH rate, patients with a prior umbilical hernia were excluded. Six patients (2.4%) developed TSH after the procedure. The incidence was higher in the recurrence group (6.7% vs. 1.9% in the no recurrence group; a difference of 4.8 pp; 95% CI −2.8 to 20.6; *p* = 0.159), but this difference was not statistically significant due to the small sample size.

When comparing the outcomes of concurrent inguinal TAPP and umbilical hernioplasty with isolated inguinal TAPP (Table 8), a higher inguinal hernia recurrence rate was observed in the TAPP + umbilical hernioplasty group, although this difference was not statistically significant (difference 1.8 pp; 95% CI −7.9 to 15.3; *p* = 0.685).

When comparing bilateral and unilateral inguinal TAPP (Table 9), a statistically significant difference was observed in the number of patients who underwent umbilical hernia repair concurrently with bilateral inguinal TAPP (16.9% in bilateral TAPP vs. 8.3% in unilateral TAPP; *p* = 0.029).

One of 113 (0.9%) patients reported TSH after bilateral inguinal TAPP, while five of 132 (3.8%) patients developed TSH following unilateral inguinal TAPP (*p* = 0.222). Furthermore, patients with a history of umbilical hernia were excluded from the TSH analysis.

As previously noted, Surgeon A consistently performs fascial closure for the midline supraumbilical 10 mm incision, whereas Surgeon B rarely does. Surgeon A operated on 130 patients, and Surgeon B on 151 (Table 10), with no significant differences in demographic and clinical characteristics. There was a significant difference in the distribution of TAPP procedure types between the surgeons, with Surgeon A performing more bilateral procedures (56.9% vs. 41.1%; difference 15.9 pp, 95% CI 4.3 to 27.4) and fewer right-sided procedures (23.8% vs. 37.1%; difference −13.2 pp, 95% CI −23.9 to −2.6). The difference in left-sided procedures was small and not statistically significant (19.2% vs. 21.9%; difference −2.6 pp, 95% CI −12.1 to 6.8). A higher proportion of patients in the Surgeon A group had an umbilical hernia before the TAPP procedure (19.2% vs. 7.3%; difference 11.9 pp, 95% CI 4.0 to 19.9; *p* = 0.003). Surgeon A had a lower TSH rate (1.0%) compared to Surgeon B (3.6%), although without a statistically significant difference (*p* = 0.242).

In the patient-level analysis (Table 11), recurrence was reported by 35 of 281 patients (12.5%) during follow-up. Multivariable logistic regression showed that previous hernia repair was the only factor independently associated with recurrence (aOR 2.22, 95% CI 1.04–4.72; *p* = 0.039). Age (aOR 0.995, 95% CI 0.971–1.020; *p* = 0.681), male sex (aOR 0.61, 95% CI 0.16–2.31; *p* = 0.468), BMI (aOR 0.98, 95% CI 0.87–1.10; *p* = 0.743), bilateral TAPP (aOR 1.13, 95% CI 0.54–2.37; *p* = 0.744), and operating surgeon (aOR 0.93, 95% CI 0.45–1.90; *p* = 0.833) were not independently associated with recurrence.

In a sensitivity analysis (Supplementary Table S3) that also included concomitant umbilical hernia repair, the association between prior hernia repair and recurrence remained significant (aOR 2.24, 95% CI 1.05–4.78; *p* = 0.037), while concomitant umbilical hernia repair was not associated with recurrence (aOR 1.29, 95% CI 0.44–3.77; *p* = 0.643).

## 4. Discussion

Recurrence after inguinal hernioplasty is multifactorial, involving a combination of surgical technique, the patient’s biology, and postoperative lifestyle factors. In our study, the patient-reported inguinal recurrence rate was 10.6% after a mean follow-up of 60 months (range 35–80). The only statistically significant risk factor was prior hernia repair, with 34.1% in the recurrence group compared to 17.2% in the no-recurrence group (*p* = 0.007). In multivariable logistic regression, previous hernia repair was the only factor independently associated with recurrence. There was no significant difference in recurrence rates after concurrent TAPP and umbilical hernia repair (12.1%) versus isolated TAPP (10.3%; *p* = 0.685). Our results align with a meta-analysis, which also identified surgery for recurrent inguinal hernia as a significant risk factor for recurrence [14]. This recurrence rate is notably higher than the commonly reported range of 1% to 4.3% [7,15,16]. The difference may stem from the long follow-up period in our study, the relatively high proportion of patients with prior hernia repairs, and the patient-reported design. In our study, recurrence was assessed solely through telephone interviews with patients, with no clinical or ultrasound examinations performed. As a result, some recurrences might have been missed if these tests had been conducted. During the follow-up, a subset of patients underwent surgery for hernia recurrence after TAPP, making these recurrences objective; however, this number was too small for further statistical analysis. In a randomized clinical trial comparing 5-year recurrence rates after Shouldice and TAPP repair, the TAPP recurrence rate was 6.6%, which aligns with our findings, especially given that only patients with unilateral primary hernias were included and follow-up was based on clinical examination [8]. In another patient-reported study, the recurrence rate was 8.1%, while clinical examination revealed a recurrence in 2.4% of patients after 34 months of follow-up [17]. These findings suggest that further research, including patients with prior repairs and bilateral hernias, as well as longer follow-up, may produce more accurate recurrence rates.

Surgeon A’s group showed a 2.5 times higher incidence of umbilical hernias. This can partly be explained by the higher proportion of patients with bilateral inguinal hernias in Surgeon A’s group (56.9% vs. 41.1% in Surgeon B’s group; *p* = 0.020), since shared genetic predisposition is proven for different types of abdominal hernias [18] and other acquired risk factors, such as increasing age and abdominal pressure. Another reason could be that Surgeon A performed umbilical hernioplasties on very small and clinically ‘silent’ umbilical hernias, found during surgery on a relaxed, intubated patient.

Another issue is midline supraumbilical TSH, reported in 2.45% of patients. To our knowledge, only one study examined the incidence and risk factors for umbilical TSH after TAPP repair [11]. Their results showed a higher TSH rate after TAPP at 3.9%. This could be due to a high proportion of synchronous umbilical hernia repairs in the study (32.9%), whereas we did not include patients with synchronous umbilical hernia repair in the TSH analysis. They state that ‘patients who underwent umbilical repairs showed a threefold increase in TSH rate,’ but we disagree with the terminology. The authors, as well as we, place a 10 mm trocar through the umbilical hernia defect, and after trocar removal, an umbilical hernioplasty is performed. Therefore, it is not TSH but a recurrent umbilical hernia.

Risk factors for inguinal hernia may also be risk factors for midline supraumbilical TSH. For example, the overall incidence of TSH at various trocar insertion sites reaches 24.5% in bariatric surgery cases (pooled incidence from studies in which TSH is the primary outcome) [19]. An even higher incidence of 34% was observed when follow-up ultrasonography was performed [20], indicating a concerningly high rate. Based on our study results, it remains unclear whether fascial closure at the supraumbilical position prevents TSH. Surgeon A, who always performed fascial closure for the supraumbilical 10 mm incision, showed a lower TSH rate (0.95%) compared to Surgeon B (3.57%), but without a statistically significant difference, likely due to the small sample size. Despite the statistics, a trend toward a lower TSH rate after fascial closure is noted. Therefore, we recommend fascial closure, especially in bilateral hernias that suggest collagen abnormalities or deficits. We used 10 mm bladed trocars for midline supraumbilical insertion at an angle of 70–90°. Lower TSH rates were observed with non-bladed trocars compared to bladed trocars for 5 mm, 10 mm, and 12 mm ports. Also, midline trocar insertion sites have a higher TSH rate [21]. An animal study using 12 mm conical bladeless trocars confirms these findings and introduces a new factor: smaller fascial defects were observed when trocars were inserted at a 45° angle [22]. Studies suggest that an umbilical hernia is a risk factor for TSH near the umbilicus [11,23]. We should interpret these findings cautiously, as we used the umbilical hernia, when present, as the 10 mm trocar insertion site.

Trocar size is associated with TSH risk, particularly at sites with 10 mm or larger trocars. Most TSHs also occur at midline trocar sites, particularly at the umbilical area, likely due to the structural weakness of that part of the abdominal wall [21,24]. Given the trend toward lower TSH rates with fascial closure, we recommend a 10 mm midline supraumbilical incision with fascial closure, especially for patients with multiple risk factors for abdominal wall hernia. The number of cases is small, so definitive conclusions cannot be made; additionally, this group has some collagen issues, making broad recommendations inappropriate. This study could lead to further multicenter studies with definitive findings.

The main limitations of this study are its retrospective, patient-reported design, which introduces recall bias. Patients were interviewed by phone instead of being examined in person by a healthcare provider, which may have affected the results. Additionally, the presence of diastasis recti was not documented, leading to a higher rate of concomitant umbilical hernia or supraumbilical TSH [11]. Moreover, Surgeon B rarely placed a fascial suture at the supraumbilical trocar site. To accurately compare with Surgeon A, no patient in the Surgeon B group should have received a fascial suture. For these reasons, the analysis cannot determine the independent effect of fascial closure on TSH occurrence.

## 5. Conclusions

The findings of this study show a 10.6% patient-reported recurrence rate of inguinal hernia and a 2.4% TSH rate in the umbilical region following TAPP. Performing umbilical hernioplasty simultaneously with TAPP repair was not associated with a statistically significant increase in inguinal hernia recurrence; however, the observed trend and the small sample size highlight the need for further prospective, multicenter studies. This research provides valuable insights into preventing TSH in laparoscopic surgery. Although our analysis did not find a statistically significant difference in TSH rates with or without fascial closure of the 10 mm midline supraumbilical ports, there was a noticeable trend toward lower rates with fascial closure using a single absorbable (polyglactin) suture 0 with a 5/8 round needle (1.0% compared to 3.6% without closure). Therefore, the findings support considering routine fascial closure of 10 mm midline incisions, with a possible benefit of reducing TSH risk, pending confirmation in larger prospective or multicenter studies.

## Figures and Tables

**Table 1:** General, demographic, and clinical data for patients with surgically treated inguinal hernias.

	All Patients	Recurrence	No Recurrence	*p*
Number of patients, *n* (%; 95% CI)	281	35 (12.5; 9.0–16.8)	246 (87.5; 83.2–90.9)	
Follow-up (months), median (IQR)	60.0 (35.0–80.0)	59.0 (40.0–80.0)	61.0 (33.0–80.0)	0.895
Age, median (IQR)	58.0 (46.0–68.0)	58.0 (40.0–67.0)	58.0 (46.0–68.0)	0.699
Female, *n* (%)	17 (6.1)	3 (8.6)	14 (5.7)	0.454
BMI, median (IQR)	26.0 (24.3–28.2)	26.12 (24.4–28.6)	26.01 (24.3–28.1)	0.932
ASA I, *n* (%)	68 (24.2)	7 (20.0)	61 (24.8)	0.296
ASA II, *n* (%)	174 (61.9)	21 (60.0)	153 (62.2)	
ASA III, *n* (%)	39 (13.9)	7 (20.0)	32 (13.0)	
Smoker, *n* (%; 95% CI)	60 (21.4)	7 (20.0; 10.1–35.9)	53 (21.5; 16.9–27.1)	0.835
Diabetes, *n* (%; 95% CI)	16 (5.7)	3 (8.6; 3.0–22.4)	13 (5.3; 3.1–8.8)	0.431
Positive family history ^1^, *n* (%; 95% CI)	84 (29.9; 24.8–35.5)	12 (34.3; 20.8–50.8)	72 (29.3; 23.9–35.2)	0.560

^1^ for inguinal hernias (see Study design). CI: confidence interval; IQR: interquartile range; BMI: body mass index; ASA: American Society of Anesthesiologists.

**Table 2:** Level of physical activity.

	All Patients	Recurrence	No Recurrence	*p*
Low physical activity, *n* (%)	51 (18.2)	6 (17.1)	45 (18.3)	0.964
Intermediate physical activity, *n* (%)	146 (52.0)	19 (54.3)	127 (51.6)	
High physical activity, *n* (%)	84 (29.9)	10 (28.6)	74 (30.1)	

**Table 3:** Distribution of laterality of TAPP procedures.

	All Patients	Recurrence	No Recurrence	*p*
TAPP bilateral, *n* (%; 95% CI)	136 (48.4; 42.6–54.2)	17 (48.6; 33.0–64.5)	119 (48.4; 42.2–54.6)	0.827
TAPP right, *n* (%; 95% CI)	87 (31.0; 25.9–36.6)	12 (34.3; 20.8–50.8)	75 (30.5; 25.1–36.5)	
TAPP left, *n* (%; 95% CI)	58 (20.6; 16.3–25.8)	6 (17.1; 8.1–32.7)	52 (21.1; 16.5–26.7)	

CI: confidence interval; TAPP: transabdominal preperitoneal (repair).

**Table 4:** Time to return to work and postoperative complications.

	All Patients	Recurrence	No Recurrence	*p*
Return to work (weeks), median (IQR)	3.0 (1.5–5.6)	3.0 (1.5–6.0)	3.0 (1.5–6.0)	0.927
Seroma/Hematoma, *n* (%)	52 (18.5)	5 (14.3)	47 (19.1)	0.492
Shoulder pain, *n* (%)	4 (1.4)	1 (2.9)	3 (1.2)	0.414
Groin pain, *n* (%)	34 (12.1)	5 (14.3)	29 (11.8)	0.590
Abdominal pain, *n* (%)	6 (2.1)	0 (0.0)	6 (2.4)	1.000

IQR: interquartile range.

**Table 5:** Inguinal hernia recurrence.

	All Hernias	Recurrence	No Recurrence	*p*
Total number of hernias, *n* (%; 95% CI)	417	44 (10.6; 8.0–13.9)	373 (89.4; 86.1–92.0)	
Right-sided hernias, *n* (%; 95% CI)	223	26 (11.7; 8.1–16.5)	197 (88.3; 83.5–91.9)	0.430
Left-sided hernias, *n* (%; 95% CI)	194	18 (9.3; 5.9–14.2)	176 (90.7; 85.7–94.0)	

IQR: interquartile range.

**Table 6:** Previously repaired hernias and recurrence.

	All Hernias	Recurrence	No Recurrence	*p*
PR hernias, *n* (%; 95% CI)	79 (18.9; 15.5 to 23.0)	15 (34.1; 20.6 to 49.3)	64 (17.2; 13.7 to 21.3)	0.007
Right-sided PR hernias, *n* (%; 95% CI)	48 (21.5; 16.6 to 27.4)	11 (42.3; 25.6 to 61.1)	37 (18.8; 14.0 to 24.8)	0.006
Left-sided PR hernias, *n* (%;95% CI)	31 (16.0; 11.5 to 21.8)	4 (22.2; 9.0 to 45.2)	27 (15.3; 10.8 to 21.4)	0.497

PR: previously repaired (hernia).

**Table 7:** Concurrent TAPP and umbilical hernia repair and TSH incidence.

	All Patients	Recurrence	No Recurrence	*p*
Umbilical hernia + TAPP, *n* (%; 95% CI)	35 (12.5; 9.1–16.8)	5 (14.3; 6.3–29.4)	30 (12.2; 8.7–16.9)	0.784
TSH after TAPP, *n* (%; 95% CI)	6 (2.4; 1.1–5.2)	2 (6.7; 1.8–21.3)	4 (1.9; 0.7–4.7)	0.159

TAPP: transabdominal preperitoneal (repair); TSH: trocar site hernia.

**Table 8:** Concurrent TAPP and umbilical hernia repair outcomes.

	Umbilical Hernia + TAPP	TAPP	*p*
Inguinal hernia recurrence, *n* (%; 95% CI)	7 (12.1; 6.0–22.9)	37 (10.3; 7.6–13.9)	0.685
Total number of inguinal hernias, *n*	58	359	

TAPP: transabdominal preperitoneal (repair).

**Table 9:** Concurrent umbilical hernia repair and comparison of bilateral vs. unilateral TAPP, along with TSH incidence after bilateral vs. unilateral TAPP.

	All Patients	Bilateral TAPP	Unilateral TAPP	*p*
Umbilical hernia + TAPP, *n* (%)	35 (12.5)	23 (16.9)	12 (8.3)	0.029
TSH after TAPP, *n* (%)	6 (2.4)	1 (0.9)	5 (3.8)	0.222

TAPP: transabdominal preperitoneal (repair); TSH: trocar site hernia.

**Table 10:** Surgeon A and Surgeon B comparison.

	Surgeon A	Surgeon B	*p*
Number of patients, *n* (%)	130 (46.3)	151 (53.7)	
Follow-up (months), median (IQR)	63.5 (35.0–79.3)	59.0 (33.0–81.0)	0.775
Age, median (IQR)	57.50 (46.8–67.0)	59.00 (46.0–69.0)	0.649
Female, *n* (%)	7 (5.4)	10 (6.6)	0.664
BMI, median (IQR)	26.1 (24.4–28.4)	26.00 (24.2–27.8)	0.539
ASA I, *n* (%)	30 (23.1)	38 (25.2)	0.826
ASA II, *n* (%)	83 (63.8)	91 (60.3)	
ASA III, *n* (%)	17 (13.1)	22 (14.6)	
Smoker, *n* (%;95% CI)	28 (21.5; 15.1–28.7)	32 (21.2; 15.0–28.6)	0.944
Diabetes, *n* (%;95% CI)	8 (6.2; 3.2–11.7)	8 (5.3; 2.7–10.1)	0.758
Low physical activity, *n* (%)	23 (17.7)	28 (18.5)	0.952
Intermediate physical activity, *n* (%)	67 (51.5)	79 (52.3)	
High physical activity, *n* (%)	40 (30.8)	44 (29.1)	
Positive family history, *n* (%;95% CI)	37 (28.5; 21.4–36.8)	47 (31.1; 24.3–38.9)	0.627
TAPP bilateral, *n* (%;95% CI)	74 (56.9; 48.4–65.4)	62 (41.1; 33.3–48.8)	0.020
TAPP right, *n* (%;95% CI)	31 (23.8; 16.5–31.2)	56 (37.1; 29.4–44.8)	
TAPP left, *n* (%;95% CI)	25 (19.2; 12.5–26.0)	33 (21.9; 15.3–28.4)	
Total number of hernias, *n* (%)	204 (48.9)	213 (51.1)	
Primary recurrent hernia, *n* (%;95% CI)	37 (18.1; 13.5–24.0)	42 (19.7; 14.9–25.6)	0.680
Return to work (weeks), median (IQR)	3.5 (2.0–6.0)	4.0 (2.0–6.0)	0.113
Seroma/Hematoma, *n* (%)	35 (27.0)	17 (11.3)	<0.001
Shoulder pain, *n* (%)	1 (0.0)	3 (2.00)	0.627
Groin pain, *n* (%)	15 (11.5)	19 (12.6)	0.789
Abdominal pain, *n* (%)	2 (1.5)	4 (2.6)	0.689
Patients with recurrence, *n* (%;95% CI)	17 (13.1; 8.0–20.6)	18 (11.9; 7.4–18.3)	0.770
Recurrence of the hernia, *n* (%;95% CI)	23 (11.3; 7.3–16.9)	21 (9.9; 6.5–14.6)	0.638
Umbilical h. before TAPP, *n* (%;95% CI)	25 (19.2; 12.5–26.0)	11 (7.3; 4.1–12.6)	0.003
Umbilical h. + TAPP, *n* (%;95% CI)	24 (18.5; 12.7–26.0)	11 (7.3; 4.1–12.6)	0.005
TSH after TAPP, *n* (%;95% CI)	1 (1.0; 0.0–4.1)	5 (3.6; 1.0–7.4)	0.242

CI: confidence interval; IQR: interquartile range; BMI: body mass index; ASA: American Society of Anesthesiologists; TAPP: transabdominal preperitoneal (repair); TSH: trocar site hernia.

**Table 11:** Multivariable logistic regression analysis of factors associated with patient-level recurrence.

Variable	Adjusted OR	95% CI	*p*
Age (per year)	0.995	0.971–1.020	0.681
Male sex	0.612	0.162–2.306	0.468
BMI (kg/m^2^)	0.981	0.874–1.101	0.743
PR hernia	2.217	1.042–4.716	0.039
Bilateral TAPP	1.131	0.540–2.366	0.744
Surgeon B vs. Surgeon A	0.925	0.450–1.903	0.833

BMI: body mass index; PR: previously repaired (hernia); TAPP: transabdominal preperitoneal repair.

## Data Availability

We have not generated or reported research data.

## Supplementary Materials

The following supporting information can be downloaded at: https://www.mdpi.com/article/10.3390/jcm15083083/s1, Table S1: Structured telephone interview; Table S2: Patients’ data from electronic medical records; Table S3: Sensitivity multivariable logistic regression analysis, including concomitant umbilical hernia repair.

## Abbreviations

The following abbreviations are used in this manuscript:

TAPP	Transabdominal preperitoneal (repair)
TSH	Trocar site hernia
UHC	University Hospital Centre
TEP	Totally extraperitoneal (repair)
PR	Previously repaired (hernia)
BMI	Body mass index
ASA	American Society of Anesthesiologists
IQR	Interquartile range
CI	Confidence interval
