# Supplementary material for: Does Inguinal TAPP Repair Increase the Rate of Midline Supraumbilical Trocar Site Hernia?—A Single-Center Retrospective Study
Source: J Clin Med. 2026 Apr 17;15(8):3083. doi: 10.3390/jcm15083083 (PMC13117957; doi:10.3390/jcm15083083)
Supplement: Supplementary file 1 [file jcm-15-03083-s001.zip › Table S1.pdf]

Name and surname:

Contact (phone number):

Date (of the interview):

Height and weight:

1. How long after the TAPP procedure did you return to work/your usual activities (for unemployed or retired patients)?
2. Did you have any complications after the operation?
   - IF YES:
     3. Which complications and when did they occur?
4. Did you experience a recurrence of the hernia that was repaired?
   - IF YES:
     5. When did the symptoms of recurrence begin? *Express in weeks or months after the operation.*
     6. Was the hernia operated on again?
        - IF YES:
          7. When were you re-operated (date)?
          8. Which surgical technique was used?
          9. Do you have a recurrence of the operated hernia after that re-operation?

10. How would you rate the level of your physical activity in daily life? *Employed patients were asked to base their assessment on the type of job they had, and unemployed patients were asked to compare the proposed job titles with their daily activities.*

   0 – unknown/cannot assess

   1 – low: office worker, teacher, hairdresser, security guard, architect, scientist

   2 – moderate: shop assistant, factory worker, auto mechanic, cook

   3 – high: farmer, nurse, construction worker, waiter, warehouse worker, driver

11. Has anyone in your family, like parents or siblings, had an inguinal hernia?
