# Supplementary material for: Does Inguinal TAPP Repair Increase the Rate of Midline Supraumbilical Trocar Site Hernia?—A Single-Center Retrospective Study
Source: J Clin Med. 2026 Apr 17;15(8):3083. doi: 10.3390/jcm15083083 (PMC13117957; doi:10.3390/jcm15083083)
Supplement: Supplementary file 1 [file jcm-15-03083-s001.zip › Table S3.pdf]

Table S3. Sensitivity multivariable logistic regression analysis, including concomitant umbilical hernia repair

Variable	Adjusted OR	95% CI	p
Age (per year)	0.995	0.970-1.019	0.668
Male sex	0.615	0.163-2.318	0.473
BMI (per kg/m²)	0.977	0.870-1.098	0.698
Previous hernia repair (any)	2.242	1.052-4.779	0.037
Bilateral TAPP	1.105	0.525-2.328	0.792
Surgeon B vs Surgeon A	0.952	0.458-1.982	0.896
Umbilical repair + TAPP	1.289	0.441-3.770	0.643

Sensitivity analysis of the patient-level multivariable logistic regression model, additionally including concomitant umbilical hernia repair. Results are presented as adjusted odds ratios (ORs) with 95% confidence intervals (CIs). BMI, body mass index; TAPP, transabdominal preperitoneal repair.
